# Is dogs’ heritable performance in socio-cognitive tasks truly social?

**DOI:** 10.3758/s13420-021-00498-x

**Published:** 2022-01-14

**Authors:** Giulia Cimarelli, Friederike Range

**Affiliations:** Domestication Lab, Konrad Lorenz Institute of Ethology, University of Veterinary Medicine, Vienna, Savoyenstraße 1a, A-1160 Vienna, Austria

## Abstract

Recently, Bray et al. (2021) showed that behavioural performance in cognitive tasks involving humans is highly heritable in dog puppies. Although the paper shows substantial heritability of specific behavioural traits, the absence of control conditions does not allow for strong support of the authors’ claim that the cognitive performance they measured represents a special sensitivity to human cooperative-communicative acts.

## Main text

In a recently published paper, Bray et al. (2021) claim (1) heritability of human-directed social behaviours and (2) emergence of social skills early in development without extensive socialization or training in dog puppies. They state that these results have important implications related to the domestication process and to what traits were potentially under selection during dogs’ evolutionary history (Bray et al., 2021). They tested 375 puppies on five social-cognitive tests: human interest, unsolvable task, and gesture following tasks (communicative marker and arm pointing). The puppies performed above chance from the first test trial on and showed no improvement across test trials in gesture-following tasks, and spent time gazing at the experimenter when she or he was speaking in “dogerese” to the puppy. More than 40% of the variance of two behavioural measures (success in the pointing task and gazing at the human in the human interest task) was attributable to genetic factors, while heritability of the performance in the other three tasks was considerably lower (<20%). The authors conclude that puppies are highly sensitive and receptive to diverse communicative signals from humans, including referential gestures and speech, and that variation in these traits is under strong genetic control. Thanks to the large sample size and the use of a population with known pedigree, the paper offers interesting insights on the heritability of behaviours, advancing our knowledge regarding their genetic basis.

### Heritability of human-directed social behaviour?

One of the basic assumptions of the paper is that dogs exhibit similarities to humans in their sensitivity to cooperative-communicative cues’ implying that similar cognitive mechanisms underlie dogs’ and human infants’ inclination towards cooperative and communicative skills. However, there are a number of methodological issues that warrant caution in choosing this interpretation.

The highest heritability was found in the pointing and human-interest tasks. The version of the pointing gesture used (sustained proximal instead of momentary distal pointing) allows the animals to solve the task by local enhancement since the finger is close to the target while the animal makes its choice (Miklósi & Soproni, 2006). When attending to the communicative gesture, wolf puppies actually perform similarly to dog puppies in this task (Gácsi et al., 2009), as do other animal species (e.g., goats; see discussion in Bray et al., 2021), suggesting that a special understanding of the cooperative-communicative nature of the human gesture may not be necessary to solve the task. Furthermore, the pointing task was always conducted after the communicative marker test. The puppies performed slightly worse in the pointing than in the marker test despite the fact that both tasks were very similar and should have been solved by the same sensitivity to human cooperative-communicative cues. In fact, in both cases, ostensive cues accompanied the gestures and the arm movements were similar, with the only difference that in the communicative marker task a yellow block was left next to the baited cup, while in the pointing task, the extended finger remained close to the baited cup until the subject made a choice. The slightly larger distance between finger and baited cup versus marker and baited cup might require more attention, and thus, animals able to sustain their attention for longer might have been more successful. The fact that the order of the tasks was not counterbalanced might have increased this effect due to tiredness of the puppies in the pointing task. Variation in sustained attention could therefore explain why heritability was only found for the pointing task.

The only other highly heritable behaviour was the duration of gazing towards the only stimulus present during the human interest task. In this test, the dog was left alone in an enclosed area with nothing to do. The only stimulus in the area was the talking human, allowing for the possibility that the saliency of the stimulus drew the attention of the puppy rather than the social nature of the stimulus. Control conditions with animated objects or at least an alternative option to spend time with (e.g., an object to play with) would have resolved this uncertainty.

Accordingly, whether the two tasks measured heritability of social factors rather than, for example, attentiveness or some personality aspect like boldness, remains unclear. The original test battery comprised 14 social and nonsocial tests (Bray et al., 2020), but the authors only investigated the heritability of single behaviours separately, and only in the “social” tasks, not reporting whether the performance in the different subtests was correlated. This analysis would have helped to identify possible underlying mechanisms/traits (e.g., attentiveness, boldness) affecting dogs’ performance and their potential specificity in relation to the social/human-directed domain. However, an even more convincing approach would be a systematic investigation of what each subtest actually measures, including specific control conditions to help determine the trait being considered (e.g., using a nonsocial stimulus in the interest task).

To conclude, although we agree that there is good evidence of heritability for puppies’ propensity to follow the pointing task and looking at the human, it is unclear whether these tasks actually measure social abilities or some other cognitive mechanism or behavioural trait.

### Early emergence of social skills without extensive socialization or training in dog puppies?

The authors claim that the present study demonstrates that dogs’ social skills emerge early in development without extensive socialization and learning, which is stated as further support for the claim of a genetic basis for these behaviours. However, studies have shown that experience and learning play an important role in the animals’ success in such tasks (e.g., Dorey et al., 2010). Indeed, although still very young, the puppies had quite extensive experiences with humans before being tested. They were all bred and socialized specifically to become assistance dogs, and their breeders were requested to engage in early neural stimulations (ENS) starting at Day 5. Accordingly, the puppies had contact with humans every day, which might have included play and other interactions (Dr. Emily Bray, personal communication). Hence, even if they had not experienced any formal training, all puppies were quite extensively socialized and had many opportunities to learn about humans and their communication. Second, as mentioned above, the puppies might have learned across tasks in the test battery. Acknowledging these facts would not mean that past selection pressure did not play a role in shaping dogs’ potential social skills. In fact, domestication might have equipped dogs with genetic predispositions and learning preferences that allow them to interact with humans in a different way than other species (including wolves).

## Conclusions

The heritability analyses on such a large sample to understand which cognitive traits have a genetic basis, and thus what domestication could have selected for in dogs, is important and valuable. However, while the authors argue that their results are robust independently from the mechanisms underlying dogs’ performance in these tasks, we think that without a critical consideration of the mechanisms, alternative interpretations are not ruled out. Just because the experimental setting includes a human does not mean that these skills are social (and even specific to the interaction with humans). We thus would like to encourage researchers to dissect the tasks currently used for investigating social cognition to uncover the underlying mechanisms necessary to solve them. In line with this, we urge researchers to not only test dogs with humans, but to also carry out appropriate controls with nonanimate objects, animate objects, or even partners belonging to different species, to disentangle what behaviours (if any) are specific to the interaction with social beings and/or with humans.
